# The Modulation of NADPH Oxidase Activity in Human Neutrophils by Moroccan Strains of *Leishmania major* and *Leishmania tropica* Is Not Associated with p47^phox^ Phosphorylation

**DOI:** 10.3390/microorganisms9051025

**Published:** 2021-05-10

**Authors:** Hasnaa Maksouri, Dounia Darif, Jerome Estaquier, Myriam Riyad, Christophe Desterke, Meryem Lemrani, Pham My-Chan Dang, Khadija Akarid

**Affiliations:** 1 Research Team on Immunopathology of Infectious and Systemic Diseases, Laboratory of Cellular and Molecular Pathology, Faculty of Medicine and Pharmacy (FMPC), Hassan II University of Casablanca (UH2C), 20000 Casablanca, Morocco; hasnaamaksourii@gmail.com (H.M.); myriamriyad@gmail.com (M.R.); 2 Molecular Genetics and Immunophysiopathology Research Team, Health and Environment Laboratory, Aïn Chock Faculty of Sciences, UH2C, 20000 Casablanca, Morocco; douniadarif93@gmail.com; 3 INSERM U1124, Paris University, 75006 Paris, France; 4 Centre Hospitalier Universitaire (CHU) de Québec Research Center, Faculty of Medicine, Laval University, Quebec City, QC G1V0A6, Canada; 5 Faculty of Medicine of the Kremlin-Bicêtre, University Paris-Sud, 94270 Paris, France; christophe.desterke@inserm.fr; 6 Laboratory of Parasitology and Vector-Borne-Diseases, Institut Pasteur du Maroc, 20250 Casablanca, Morocco; meryem.lemrani@pasteur.ma; 7 INSERM-U1149, CNRS-ERL8252, Inflammation Research Center, 75018 Paris, France; my-chan.dang@inserm.fr; 8 Inflamex Laboratory of Excellence, Faculty of Medicine, Site Xavier Bichat, University of Paris, 75018 Paris, France

**Keywords:** *Leishmania*, neutrophils, NADPH oxidase, ROS, p47^phox^

## Abstract

Polymorphonuclear neutrophils (PMNs) are the first phagocyte recruited and infected by *Leishmania*. They synthetize superoxide anions (O_2_^−^) under the control of the NADPH oxidase complex. In Morocco, *Leishmania major* and *L. tropica* are the main species responsible for cutaneous leishmaniasis (CL). The impact of these parasites on human PMN functions is still unclear. We evaluated the in vitro capacity of primary Moroccan strains of *L. major* and *L. tropica* to modulate PMN O_2_^−^ production and p47^phox^ phosphorylation status of the NADPH oxidase complex. PMNs were isolated from healthy blood donors, and their infection rate was measured by microscopy. O_2_^−^ production was measured by superoxide dismutase–inhibitable reduction of cytochrome C. P47^phox^ phosphorylation was analyzed by Western blot using specific antibodies against Ser328 and Ser345 sites. Whereas we did not observe any difference in PMN infectivity rate, our results indicated that only *L. tropica* promastigotes inhibited both fMLF- and PMA-mediated O_2_^−^ production independently of p47^phox^ phosphorylation. *Leishmania* soluble antigens (SLAs) from both species significantly inhibited O_2_^−^ induced by fMLF or PMA. However, they only decreased PMA-induced p47phox phosphorylation. *L. major* and *L. tropica* modulated differently O_2_^−^ production by human PMNs independently of p47^phox^ phosphorylation. The inhibition of ROS production by *L. tropica* could be a mechanism of its survival within PMNs that might explain the reported chronic pathogenicity of *L. tropica* CL.

## 1. Introduction

*Leishmania* are protozoan parasites causing leishmaniases with 350 million people at risk in about 98 countries or territories, and an incidence of approximately 2 million cases per year: 0.2 to 0.4 million cases of visceral leishmaniasis (VL) and 0.7 to 1.2 million cases of cutaneous leishmaniasis (CL) per year, worldwide [1,2]. *Leishmania* parasites are dimorphic organisms that live and replicate in sandflies’ gut as flagellated forms (promastigotes) or non-flagellated forms in mammalian cells (amastigotes). In Morocco, *Leishmania tropica* and *L. major* are the main endemic species causing anthroponotic and zoonotic CL, respectively, which remain a public health problem, with 5073 cases reported to the Ministry of Health in 2016 [3,4]. These species are associated with a clinical polymorphism of cutaneous lesions in the human host with respect to the aspect, incubation period, and healing time. Previous studies have shown that the clinical manifestations of CL depend as much on the host’s immune response as on the infecting parasite’s virulence factors [5,6,7].

These parasites preferentially infect phagocytic cells, such as macrophages, polymorphonuclear neutrophils (PMNs) and dendritic cells [8]. Following the bite of an infected sandfly, PMNs are the first phagocyte lineage recruited that then deliver the parasites to macrophages [9,10,11]. *Tacchini Cottier et al* showed that early recruitment of PMNs at the site of the infection contributes to the susceptibility of BALB/c mice to *L. major* infection compared to C57BL/6 resistant mice [12]. It is likely that PMNs play a dual protective and permissive role shortly after promastigote infection by reducing the incoming parasite burden and subsequently facilitating the safe passage of surviving parasites to naïve host cells [13,14].

Pathogens are killed by phagocytes through different mechanisms such as the generation of superoxide anion (O_2_^−^), the precursor of other highly toxic reactive oxygen species (ROS) such as hydrogen peroxide (H_2_O_2_), hydroxyl radical (^•^OH) and hypochlorous acid (HOCl). Recently, we reported that *L. major* and *L. tropica* modulated the production by macrophages of nitric oxide (NO) in a different manner [15]. However, controversial results have been reported regarding ROS production by *Leishmania*-infected PMNs [16,17]. Early recruitment of PMNs at the site of infection and production of ROS could contribute to the resolution or aggravation of the pathology according to species and strains. Some species are sensitive to PMNs’ ROS production, reducing the intracellular parasite load and allowing for the infection to resolve [6,18,19]. Conversely, other species (*L. infantum and L. braziliensis*) would be able to develop resistance to ROS production, which would lead to exacerbation of the disease [16,20].

Superoxide anion is produced by a multicomponent enzyme called NADPH oxidase, which is unassembled and inactive in resting cells. This enzyme assembles at the plasma or phagosomal membrane upon activation. This complex consists of a membrane-associated flavocytochrome b_558_ (comprising two subunits, gp91^phox^ and p22^phox^) and four cytosolic components (p47^phox^, p67^phox^, p40^phox^, the GTPase Rac 1 or 2). Upon PMN stimulation, p47^phox^, p67^phox^, and p40^phox^ become phosphorylated and then migrate to the membranes, together with Rac1/2 where they associate with cytochrome b_558_ to assemble the active oxidase. P47^phox^ phosphorylation on serine in the autoinhibitory region located in the C-terminal has been shown to be crucial for NADPH oxidase assembly, priming, and activation. Whereas p47^phox^ phosphorylation on Ser345 plays a key role in NADPH oxidase priming (it is a ‘ready to go’ state, preparing the NADPH oxidase for a stronger activation) [21], p47^phox^ phosphorylation on Ser328 is important for NADPH oxidase activation [22].

Considering that PMNs constitute a double-edged sword, accounting for resistance to pathogens and for tissue injury, and given the key role of ROS and NADPH oxidase in microbicidal mechanisms, the present work aims to (i) evaluate whether primary *L. tropica* and *L. major* Moroccan strains modulate O_2_^−^ production by human PMNs, and (ii) determine if p47^phox^phosphorylation is involved in this modulation. These approaches may contribute to a better understanding of human CL physiopathology.

## 2. Material and Methods

### 2.1. Ethics Statements

This work was conducted according to the principles specified in the Declaration of Helsinki and under the local ethical guidelines (Ethics Committee for Biomedical Research, Faculty of Medicine and Pharmacy, University Hassan II of Casablanca, Casablanca, Morocco; International Review Board 00002504). The strains used in this study were isolated from the dermal lesions of two patients recruited at the Department of Dermatology (Ibn Rochd University Hospital of Casablanca, Casablanca, Morocco). Patient consent (for adults) or from parents (for minors under the age of 18 years) was obtained by the dermatologist. Oral consent was obtained before samplings. In 2010, oral consent was the sole requirement imposed by the Ethics Committee in Morocco for research purposes. Blood was obtained from healthy adult donors (Établissement Français du Sang, Paris, France). All donors signed informed consent allowing the use of their blood for research purposes.

### 2.2. Leishmania Strains

*L. tropica* (MHOM/MA/2010/L02) and *L. major* (MHOM/MA/2010/L112) strains were isolated from the skin lesions of Moroccan CL patients diagnosed in the Department of Dermatology (Ibn Rochd University Hospital of Casablanca, Casablanca, Morocco). The dermal syringe-sucked fluid was collected under sterile conditions from the border of active skin lesions from each patient. They were genotyped by ITS1 PCR–HaeIII RFLP according to Mouttaki T et al. at the Parasitology Laboratory (Faculty of Medicine and Pharmacy, Casablanca, Morocco) [23].

Promastigotes were isolated in NNN biphasic medium and then grown and maintained at 26 °C in RPMI-1640 medium (Thermo Fisher Scientific, Les Ulis, France) supplemented with 10% heat-inactivated fetal calf serum (Gibco, Les Ulis, France), 2 mM L-glutamine (Gibco, Les Ulis, France), 100 U/mL penicillin (Gibco, Les Ulis, France), and 100 ng/mL streptomycin (Gibco, Les Ulis, France). *L. major* and *L. tropica* promastigotes were used after 5 successive passages from the primary culture of the skin lesions.

### 2.3. Soluble Leishmania Promastigote Antigens (SLAs) Preparation

SLAs were prepared from stationary phase promastigotes of our *L. major* and *L. tropica* strains as described by Dey R et al. [24]. Briefly, the promastigotes were washed 3 times in cold sterile phosphate-buffered saline (PBS) and then adjusted to 10^8^ promastigotes/mL in PBS. The parasites were disrupted by 10 cycles of freezing (−80 °C) and thawing (37 °C) followed by ultra-sonication (20 times for a period of 30 s). SLAs were aliquoted and stored at −80 °C until use. The protein concentration was determined by the Bradford protein assay (kit Protein Assay, BioRad, Marnes-la-Coquette, France).

### 2.4. Human PMN Isolation

PMNs were isolated from the blood of healthy adult donors under LPS-free conditions by dextran (Millipore SIGMA, Lyon, France) sedimentation, followed by Ficoll density gradient centrifugation at 500× *g* for 30 min (GE Healthcare Life Sciences, Marcy-l’Étoile, France). Red blood cells were removed by hypotonic lysis.

Following isolation, PMNs were suspended in Hank’s balanced salt solution (HBSS, Sigma, St. Louis, MI, USA) containing Ca^++^, Mg^++^, and D-glucose.

### 2.5. In Vitro Infection of Human PMNs by L. major and L. tropica Promastigote Strains

The PMNs (10^6^ cells) were infected with stationary phase of *L.* *major* and *L. tropica* promastigote strains at a 5:1 ratio (5 parasites per PMN) and incubated for 20 min at 37 °C. Subsequently, PMNs were washed four times with PBS in order to remove non-ingested parasites and then re-suspended in PBS (pH 7.2). Cytospin smears from the cell suspension were stained with 10% Giemsa solution and examined microscopically to determine the PMN infection rate and intracellular parasite load. The rate of infected PMNs was calculated as follows: (Number of infected neutrophils/Total number of neutrophils) × 100. The intracellular parasite load was estimated by the mean number of intracellular amastigotes in 100 cells. The number of infected neutrophils was determined by microscopic analysis (×1000) coupled with a photographic system. The slides were read by two independent experimenters.

### 2.6. Superoxide Anion Production Assay

PMNs (10^6^ cells) were suspended in HBSS media and incubated with cytochrome C (Sigma, C7752, St. Louis, MI, USA). Cells were infected with *L. tropica* and *L. major* promastigotes at a 5:1 ratio (5 parasites per PMN) and incubated for 20 min at 37 °C. Cells were unstimulated (treated with HBSS) or stimulated with bacterial peptide formyl-Met-Leu-Phe (fMLF, 10^−7^M) (Sigma-Aldrich, F3506, St. Louis, MI, USA) or phorbolmyristateacetate (PMA, 100 ng/mL) (Sigma-Aldrich, P1585, St. Louis, MI, USA). After 8 min of incubation at 37 °C, cells were centrifuged, and the supernatants were used to measure total superoxide production as an end-point superoxide dismutase-inhibitable reduction of cytochrome C at 550 nm. ROS production was expressed as nmoles/8 min/10^6^ cells using the Beer-Lambert’s law (ΔDO = εCL, **ε**_cyt c_ = 21.1 mM^−1^cm^–1^ and L = 1 cm).

For SLAs, the absorbance was recorded after 5 min of stimulation. O_2_^−^ production was measured by the superoxide dismutase-inhibitable cytochrome C reduction at 550 nm in a dual beam recording spectrophotometer (UVIKON^®^860 spectrophotometer, KONTRON Instruments, Montigny-le-Bretonneux, France). Superoxide production was expressed as nanomoles of reduced cytochrome C. min^−1^ per 10^6^ cells (molar extinction coefficient: 21.1 mM^−1^ cm^−1^).

### 2.7. Neutrophils Viability

We also assessed the impact of *L. major* and *L. tropica* SLAs (5 µg/mL and 10 µg/mL) on PMNs viability. For this purpose, 2 × 10^6^ cells were incubated with different concentrations of *L. major* and *L. tropica* SLAs for 20 min at 37 °C. Cells viability was evaluated using the Trypan blue exclusion assay.

### 2.8. Superoxide Anion Scavenging Assay

The superoxide anion scavenging potential effect of SLAs was examined in a cell-free xanthine/xanthine oxidase assay system by following the reduction of cytochrome C at 550 nm. The reaction mixture prepared in HBSS contained 0.02 U of xanthine oxidase, and 100 μM of cytochrome C in the presence or absence of increasing SLA concentrations. Superoxide production was initiated by adding 500 μM of xanthine and the absorbance increase at 550 nm was recorded for 10 min at 37 °C in a spectrophotometer (UVIKON^®^860spectrophotometer, KONTRON Instruments, Augsburg, Germany). SOD (75 U) was used to determine reaction specificity.

### 2.9. SDS–PAGE and Western Blotting for p47^phox^ Phosphorylation Analysis

PMNs (4 × 10^6^cells) were infected with *L. major* or *L. tropica* promastigotes at a 5:1 ratio for 20 min at 37 °C, and then stimulated with fMLF (10^−7^ M) or PMA (100 ng/mL) at 37 °C. PMNs were also incubated at 37 °C for 20 min with or without increasing concentrations of SLAs. The reaction was stopped by adding 125 μL of 5× concentrated modified Laemmli sample buffer containing 12.5 mM Na_3_VO4, 25 mM NaF, 6.25 mM p-NPP, 12.5 mM EDTA, 12.5 mM EGTA, 50 μg/mL leupeptin, 50 μg/mL pepstatin and 50 μg/mL aprotinin. Samples were then denatured in boiling water (100 °C, 5 min) and stored at −80 °C until Western blotting analysis. Upon thawing, samples were sonicated twice for 5 s and subjected to 11% SDS–PAGE (equivalent of 4 × 10^5^ cells per well) using standard techniques. The separated proteins were transferred to nitrocellulose membranes. After saturation with 5% milk in Tris-buffered saline containing 0.1% Tween 20 (TBS-T 0.1%) for 1 h, the membranes were probed with an anti-phospho-Ser345-p47^phox^ (1:5000), or an anti-phospho-Ser328-p47^phox^ (1:5000), which were produced as previously described [25,26]. After overnight incubation at 4 °C and washing with TBS-Tween 0.1%, the membranes were incubated with the corresponding secondary antibody, i.e., HRP-labelled goat anti-rabbit antibody (1:10,000), or AP-labelled goat anti-rabbit antibody (1:10,000). The protein bands were revealed using enhanced chemiluminescence (Santa Cruz, Heidelberg, Germany, sc-2048) when HRP-conjugated secondary antibodies were used. For the AP-conjugated secondary antibodies, the bands were revealed by adding the AP substrate containing 0.015% of BCIP (5-bromo-4-chloro-3-indolyl phosphate) and 0.3 mg/mL of NBT (nitrobluetetrazolium) in 0.1 M carbonate buffer containing 1 mM MgCl_2_.

### 2.10. Statistical Analysis

Results are expressed as the mean ± SEM of at least three independent experiments. The protein bands in Western blots were quantified using Image J 1.43 software (Wayne Rasband, National Institutes of Health, Bethesda, MD, USA). Before performing the analysis, a Gaussian distribution by two different tests of Normality: the Shapiro-Wilk test and the Kolmogorov-Smirnov test attesting the exclusion of non-Gaussian distribution if the *p*-value was found to be higher than 0.05; the distribution of quantiles along the Henri line in Gaussian “qqplot” (quantile-quantile plot) was also verified graphically for comparison. These verifications were performed in an R software environment version 3.5.3. One-way ANOVA analysis of variance with the Tukey test for multiple comparisons was implemented using GraphPad Prism version 5.0 for Windows (GraphPad Software, San Diego, CA, USA). For any experiment design with comparison, factor decomposition was investigated by two-way ANOVA analysis (aov R function) with corresponding dot-and-wisker plots by ggplot2 graph definition in ggpubr R-package.

## 3. Results

### 3.1. Infection of Human PMNs by L. major and L. tropica Primary Strains

The *L. major* (MHOM/MA/2010/L02) and *L. tropica* (MHOM/MA/2010/L112) strains were isolated from patients living in Casablanca, a non-endemic area for CL. They were infected during a summer stay in a known endemic foci of CL due to *L. tropica* or *L. major.* The patients presented with different clinical phenotypes in terms of the number of dermal lesions (simple vs. multiple) and lesion healing time after treatment [15].

**Figure 1:**
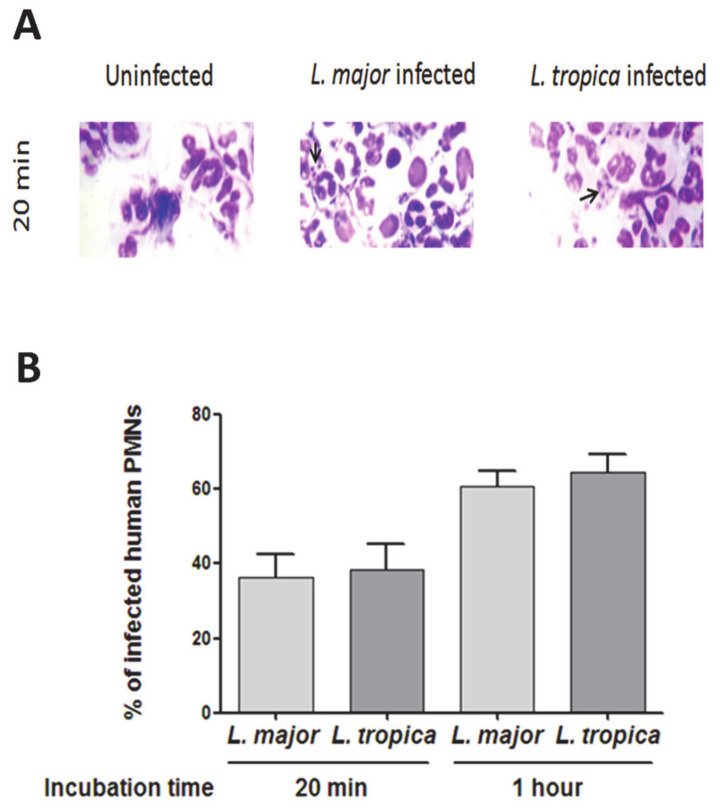
Infection rate of *L. major* and *L. tropica* primary strains in human PMNs. PMNs were cultured at 10^6^/mL, infected with *L. major* and *L. tropica* promastigotes at a 5:1 ratio, and incubated for 20 min at 37 °C. (**A**) Cytocentrifuge preparations of uninfected and infected PMNs were stained with Giemsa for light microscopy. The original photomicrographs were taken at 1000x magnification. Arrows indicate the presence of intracellular parasites in PMNs infected by *L. major* and *L. tropica* strains and (**B**) infection rates of PMNs. Results are expressed as mean ± SEM from three independent experiments, each performed in duplicate. Data were analyzed using a one-way ANOVA test with a Tukey’s *post-hoc* test for multiple comparisons.

We evaluated the infection rate of PMNs in the presence of either *L. major* or *L. tropica* primary strains. At 20 min of infection, we observed an average of 6–9 amastigotes per cell (Figure 1A). The percentages of infected PMNs were of 36.35% ± 6.27 and 38.50% ± 6.65 for *L. major* and *L. tropica* strains, respectively. These percentages reached 60.75% ± 8.11 and 64.49% ± 10.13 at 1 h post-infection (Figure 1B). Thus, no significant difference in PMN infectivity was observed with the two strains of *Leishmania* used and it was confirmed by two-way ANOVA factorial analysis (Supplemental Figure S1A, species effect *p* = 0.6092357, with significant time effect *p* = 7.325 × 10^−4^).

### 3.2. Impact of L. major and L. tropica Promastigote Strains on O_2_^−^ Production by Human PMNs

**Figure 2:**
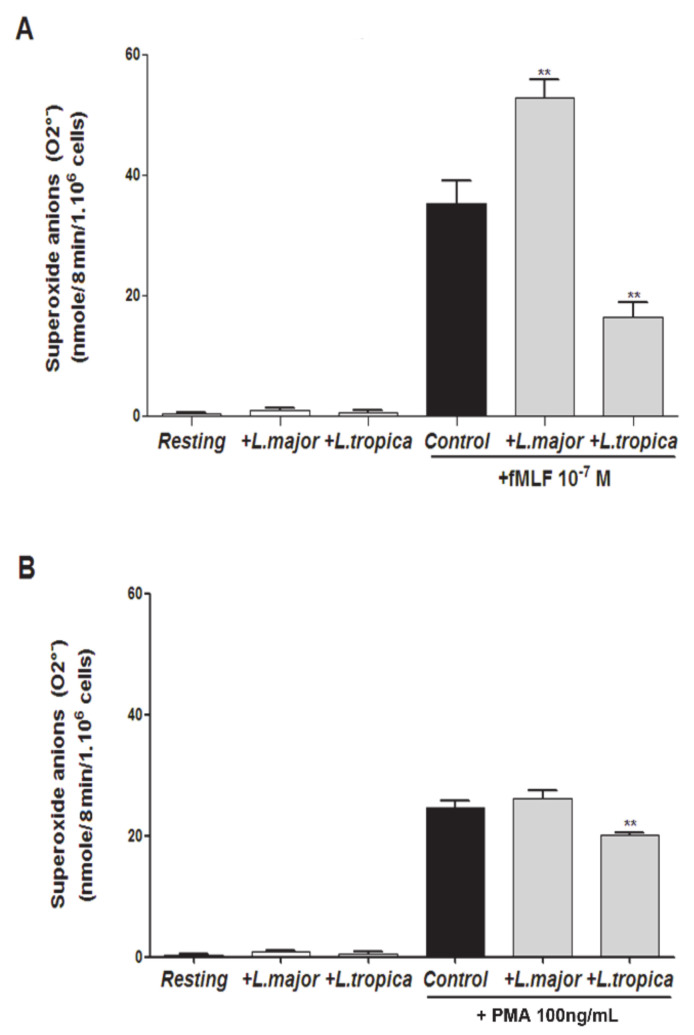
Effect of *L. major* and *L. tropica* promastigote strains on O_2_^−^ production by human PMNs. PMNs (10^6^/mL) were infected at 5:1 ratio for 20 min at 37 °C. The cells were incubated in the presence or absence of fMLF (10^−7^ M) or PMA (100 ng/mL). As a control, uninfected and nonstimulated PMNs (Resting), and uninfected and stimulated PMNs (Control) were used. Superoxide anion production was measured by the SOD-inhibitable cytochrome C reduction assay by the end-point method. The superoxide anion production was expressed as nmole/min/10^6^ cells in the presence or absence of fMLF (**A**), or PMA (**B**). Results are expressed as mean ± SEM from three independent experiments, each performed in duplicate. Data were analyzed using a one-way ANOVA test with Tukey’s *post-hoc* test for multiple comparisons. ** *p* < 0.01.

PMNs infected with stationary phase promastigotes were stimulated or not with either the bacterial formyl peptide (fMLF, 10^−7^M) or the direct PKC activator (PMA, 100 ng/mL). O_2_^−^ production was measured by an end-point SOD-inhibitable cytochrome C reduction method. Figure 2 shows that neither *L. major* nor *L. tropica* promastigotes induced O_2_^−^ production. Next, we evaluated whether they modulated O_2_^−^ production in cells stimulated with fMLF (Figure 2A) or PMA (Figure 2B), which was respectively confirmed by two-way ANOVA factorial analyses for each treatement: fMLP (Supplemental Figure S1B, treatment effect: *p* < 2.2 × 10^−16^), PMA (Supplemental Figure S1C: treatment effect: *p* < 2.2 × 10^−16^). Herein, we observed that *L. major* significantly potentiated fMLF-induced O_2_^−^ production by human PMNs (*p* < 0.01), whereas *L. tropica* significantly inhibited this production (*p* < 0.01) (Figure 2A). *L. tropica also* significantly inhibited PMA-induced O_2_^•-^ production (*p* < 0.01), whereas *L. major* had no effect (Figure 2B). These results demonstrate that primary *L. major* and *L. tropica* strains differently modulated superoxide anion production in human PMNs.

### 3.3. Impact of L. major and L. tropica SLAs on O_2_^−^ Production by Human PMNs

**Figure 3:**
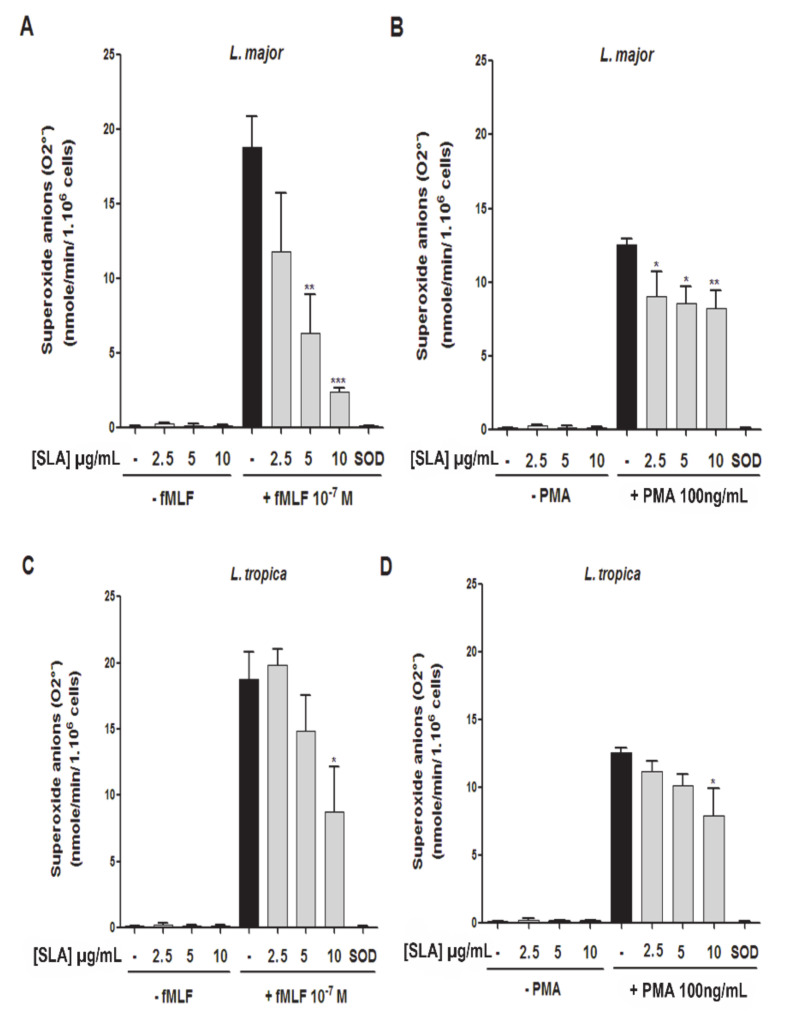
Effect of *L. major* and *L. tropica* SLAs on O_2_^−^ production by human neutrophils. PMNs were incubated (20 min at 37 °C) in the presence or absence of increasing concentrations of SLAs derived from either *L. major* (**A** and **B**) or *L. tropica* (**C** and **D**) and then incubated in the presence or absence of (**A** and **C**) fMLF (10^−7^ M) or (**B** and **D**) PMA (100 ng/mL). Superoxide anion production was measured by the SOD-inhibitable cytochrome C reduction assay. Results are expressed as mean ± SEM from 4 independent experiments, each performed in duplicate. Data were analyzed using one-way ANOVA test with Tukey’s *post-hoc* test for multiple comparisons. * *p* < 0.05; ** *p* < 0.01; *** *p* < 0.001.

We next evaluated whether soluble *Leishmania* promastigote antigens (SLAs) modulated O_2_^•^production. For this purpose, PMNs were incubated with several concentrations of *L. major* or *L. tropica* SLAs for 20 min, and then stimulated or not with fMLF (Figure 3A,C) or PMA (Figure 3B,D). The superoxide anion production was measured by the SOD-inhibitable cytochrome C reduction method. Our data showed that neither *L. major* nor *L. tropica* SLAs induced O_2_^−^ production in PMNs. However, *L. major* and *L. tropica* SLAs significantly inhibited superoxide anion generation induced by fMLF (Figure 3A,C) or PMA (Figure 3B,D). Factorial analyses by two-way ANOVA with decomposition of treatment and doses factors in case of SLA-*L. major* stimulation confirmed significant treatment effect for fMLP (Supplemental Figure S1D, *p*-value = 6.671 × 10^−7^) and also significant treatment effect for PMA (Supplemental Figure S1E, *p*-value = 5.732 × 10^−12^). The same was found in the case of SLA-*L. tropica* stimulation factorial analyses, which confirmed a significant treatment effect for fMLP (Supplemental Figure S1F, *p*-value = 6.079 × 10^−12^) and for PMA (Supplemental Figure S1G, *p*-value = 6.007 × 10^−13^).

While *L. tropica* SLA significantly inhibited O_2_^−^ production induced by fMLF or PMA at higher concentration (10 µg/mL) (Figure 3C,D), the *L. major* SLA inhibited this production in a concentration-dependent manner (2.5 µg/mL, 5 µg/mL, and 10 µg/mL) (Figure 3A,B).

**Figure 4:**
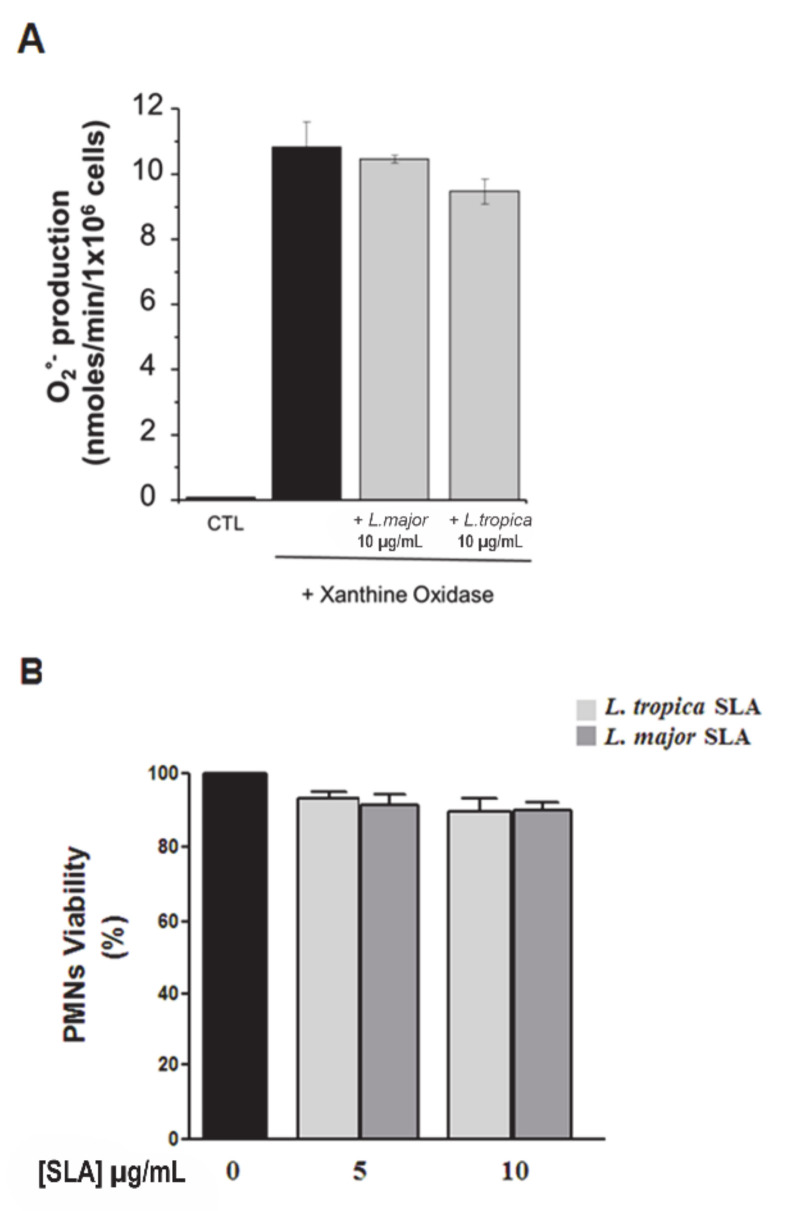
SLAs from *L. major* and *L. tropica* did not scavenge O_2_^−^ and did not affect neutrophil viability. The superoxide anion scavenging effect of SLAs was examined in a cell-free xanthine/xanthine oxidase assay. (**A**) Superoxide anion production in the presence of *L. tropica* and *L. major* SLAs was expressed as nmoles/min/10^6^ cells. CTL: control, PMN in the absence of SLA. (**B**) Percentage of viable PMNs in the presence of either *L. major* or *L. tropica* SLAs. Results are expressed as mean ± SEM from 3 independent experiments. Data were analyzed using one-way ANOVA test with Tukey’s *post-hoc* test for multiple comparisons.

Because previous studies have reported that *L. donovani* has a high effective capacity in scavenging hydroxyl radicals and superoxide anion [27,28,29], we then determined whether the inhibitory effect of *L. major* and *L. tropica* SLAs on human PMNs superoxide anion production was related to potential O_2_^−^ scavenging properties. We quantified the production of superoxide anion in a cell-free xanthine/xanthine oxidase assay in the presence or absence of SLAs. The results showed that *L. major* and *L. tropica* SLAs had no scavenging effect at the highest concentration used (10 μg/mL) (Figure 4A). Furthermore, this inhibition was not related to PMN death as assessed by a trypan blue dye exclusion assay (Figure 4B). Thus, our results showed that fMLF and PMA, in inducing O_2_^−^ production by PMNs, were differently modulated by promastigotes and SLAs.

### 3.4. Impact of L. major and L. tropica Promastigotes on the Phosphorylation Status of p47^phox^ in Human PMNs

**Figure 5:**
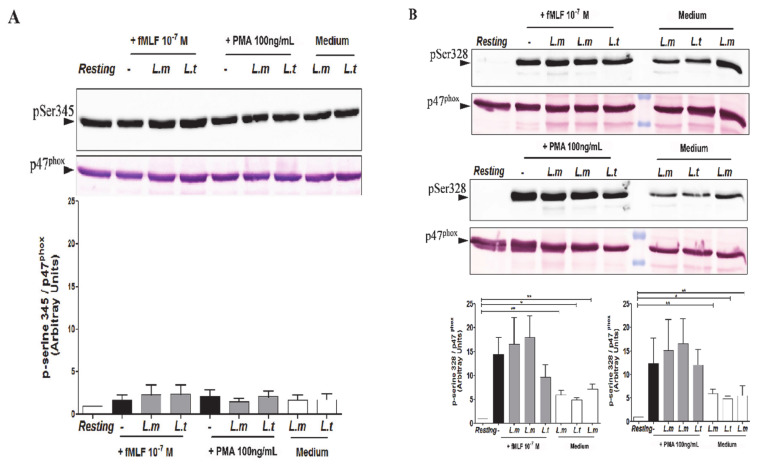
Effect of *L. major* and *L. tropica* promastigotes on the p47^phox^ phosphorylation of Ser328 and Ser345. An additional *L. major* strain was used in these experiments: (MHOM/MA /2010/L104). PMNs (4 × 10^6^) were infected at a 5:1 ratio, then stimulated with fMLF (10^−7^ M) and PMA (100 ng/mL). PMNs were infected and non-stimulated (Medium), uninfected and stimulated (−), uninfected and non-stimulated (Resting), are used as controls. Cell lysates were subjected to SDS–PAGE and analyzed by Western blot using specific rabbit polyclonal antibodies (**A**) anti-phospho-Ser345 p47^phox^ and (**B**) anti-phospho-Ser328 p47^phox^. The blots were re-probed with rabbit anti-p47^phox^ antibody as a loading control. The ratio of phospho-p47^phox^ to the total amount of p47^phox^ was quantified using Image J.Values. Results are expressed as Mean ± SEM from 5 independent experiments. Data were analyzed using a One-way ANOVA test with Tukey’s *post-hoc* test for multiple comparisons. * *p* < 0.05; ** *p* < 0.01.

Several studies have previously shown that p47^phox^ cytosolic subunit phosphorylation is a crucial event for NADPH oxidase activation [25,30], notably on Ser345 and Ser328, which are involved in NADPH oxidase priming and activation, respectively [22]. We then examined the impact of *L. major* and *L. tropica* on the phosphorylation status of p47^phox^ (Figure 5). PMNs were first infected with *Leishmania* promastigotes, and then stimulated with or without either fMLF or PMA. The phosphorylation status of p47^phox^ on Ser345 (Figure 5A) and on Ser328 (Figure 5B) was analyzed by Western blot, using specific anti-phospho-Ser345 and anti-phospho-Ser328 antibodies, as previously reported [25,31]. Our results showed that phosphorylation of p47^phox^ on Ser345 in freshly unstimulated PMNs was unchanged. fMLF and PMA stimulation had no effect on p47^phox^ phosphorylation on Ser345, as has already been shown [25]. Furthermore, our results indicated that *L. major* and *L. tropica* promastigote infection had no impact on p47^phox^ phosphorylation on Ser345 after fMLF or PMA stimulation (Figure 5A), being confirmed by factorial analysis with decomposition of the species and treatment factors (Supplemental Figure S2A, species effect *p*-value = 0.9013 and treatment effect *p*-value = 0.7902). On the contrary, we observed that fMLF and PMA stimulation induced a p47^phox^ phosphorylation on Ser328 (Figure 5B), which was confirmed by factorial analysis with decomposition of the species and treatment factors (Supplemental Figure S2B, specie effect *p*-value = 0.20316 and treatment effect *p* = 0.01772). We also observed that both *Leishmania* strains induced Ser328 phosphorylation of p47^phox^ in the absence of stimulation (Figure 5B). However, nor *L. major* or *L. tropica* promastigotes significantly inhibited p47^phox^ phosphorylation on Ser328 (Figure 5B); thus, our results indicate that *L. major* and *L. tropica* promastigotes modulated O_2_^−^ production induced by fMLF or PMA independently of p47^phox^ phosphorylation.

### 3.5. Impact of SLAs on p47^phox^ Phosphorylation in Human PMNs

**Figure 6:**
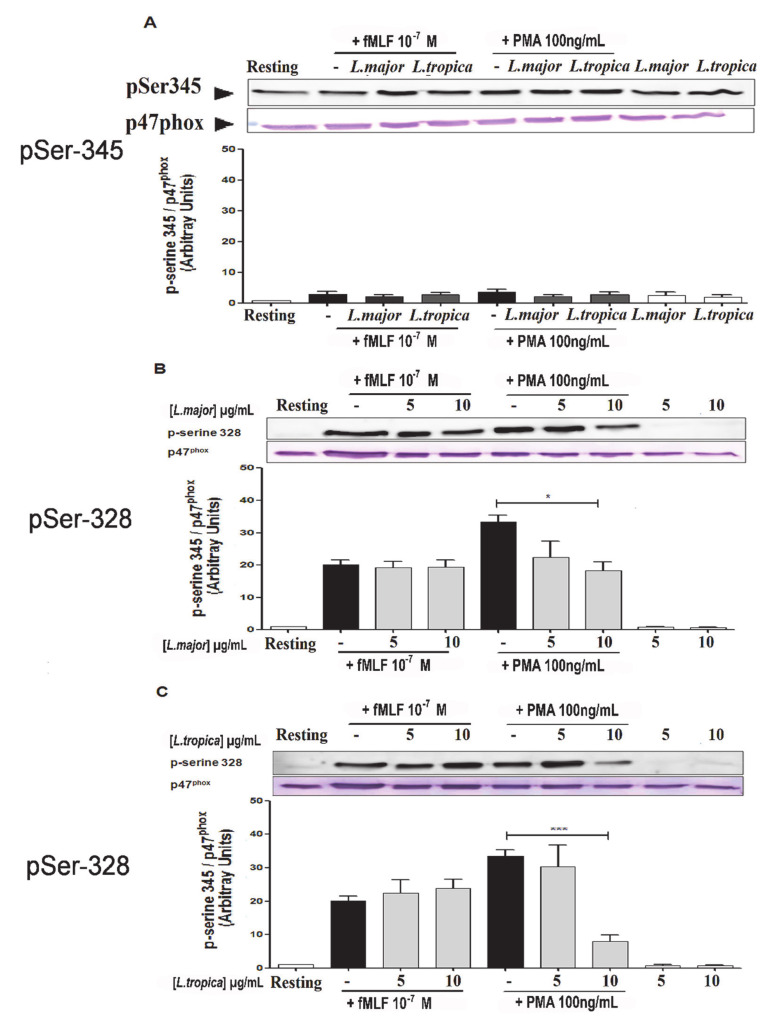
Effect of *L. major* and *L. tropica* SLAs on the p47^phox^ phosphorylation on Ser328 and Ser345. PMNs (4 × 10^6^) were incubated with SLAs at 10µg/mL (**A**)**,** 5 µg/mL and 10µg/mL (**B,C**) and then stimulated with fMLF (10^−7^ M) and PMA (100 ng/mL). PMNs were also incubated with SLA in the absence of further stimulation. As controls, PMNs were incubated in medium alone (Resting), PMNs were non infected and stimulated (−). Cell lysates were subjected to SDS–PAGE and analyzed by Western blotting using specific rabbit polyclonal antibodies (**A**) anti-phospho-Ser345 p47^phox^ and (**B,C**) antiphospho-Ser328 p47^phox^ (**B**): *L. major* SLA and **C**: *L. tropica* SLA). The blots were probed again with a rabbit anti-p47^phox^ antibody to assess loading. The ratio of phospho-p47^phox^ to the total amount of p47^phox^ was quantified using Image J 1.43u software. Values are expressed as Mean ± SEM from 6 independent experiments. Data were analyzed using one-way ANOVA test with Tukey’s *post-hoc* test for multiple comparisons. * *p* < 0.05; *** *p* < 0.001.

We then examined the impact of SLAs on the phosphorylation status of the p47^phox^ on Ser345 (Figure 6A) and Ser328 (Figure 6B,C). PMNs were incubated with SLAs (5 and 10 µg/mL), then stimulated or not with either fMLF or PMA. Our results indicated that SLAs from both *Leishmania* strains had no effect on Ser345 phosphorylation (Figure 6A), which was confirmed by two-way ANOVA factorial analysis with species and treatment effects (Supplemental Figure S2C, specie: *p*-value = 0.5424 and treatment: *p* = 0.8990). Contrary to the promastigote’s infection, SLAs did not induce the phosphorylation of p47^phox^ on Ser328 in PMNs. Furthermore, we observed that SLAs decreased the levels of p47^phox^ phosphorylated form on Ser328 mediated by PMA, a PKC activator (Figure 6B,C). Thus, at the dose of 10µg/mL, *L. tropica* SLAs reduced by 75% this amount, whereas this is reduced by only 50% in the presence of *L. major* SLAs. Interestingly, we observed no modulation of the p47^phox^ phosphorylated form on Ser328-induced by fMLF. Altogether, our results indicated that SLAs from *Leishmania* modulated the phosphorylation on Ser328 of p47^phox^, which is important for NADPH oxidase activation, which depends on the PKC pathway. For testing the effect of SLA on P-ser328 p47phox, two-way ANOVA factorial analyses performed with treatment and doses confirmed significant effect of treatments in the case of stimulation with *L. major* SLA (Supplemental Figure S2D, *p*-value = 9.545 × 10^−10^) or with *L. tropica* SLA (Supplemental Figure S2E, *p*-value =3.889 × 10^−7^).

## 4. Discussion

In Morocco, CL is a major public health problem with two main causative entities: the zoonotic form due to *L. major* and the anthroponotic form due to *L. tropica* [32]. Although macrophages are the definitive refuge for *Leishmania* into the host, neutrophils are considered by many as a transitional refuge for parasites that are able to survive the toxic extracellular environment [33,34]. Furthermore, it has been shown that PMN recruitment at the infection site contributes to host susceptibility or resistance to *Leishmania* infection [34,35]. Despite the early recruitment of PMNs following *Leishmania* infection, the impact of the parasites on human PMN functions, especially ROS production is still unclear. In this study, we evaluated whether primary strains of *L. major* and *L. tropica* and their respective SLAs modulated superoxide anion (O_2_^−^) production by human PMNs and analyzed the phosphorylation status of the p47^phox^ involved in the activation of the NADPH oxidase [25,30]. We demonstrated that both promastigote strains modulated fMLF and PMA-induced O_2_^−^ production in a different manner. Whereas *L. major* potentiated fMLF-induced O_2_^−^ production or had no effect on the levels of O_2_^−^ production induced by PMA, *L. tropica* promastigotes inhibited both. Our results also indicated that SLAs derived either from *L. major* or *L. tropica* had no impact on the basal levels of O_2_^−^ production but inhibited O_2_^−^ production induced by fMLF or PMA in a concentration-dependent manner. By analyzing the phosphorylation status of the subunit p47^phox^, our results indicated that SLAs reduced the levels of the p47^phox^ phosphorylated form on Ser328 but had no effect on Ser345 phosphorylation mediated by fMLF or PMA. This effect was more pronounced when using SLAs derived from *L. tropica* compared to those derived from *L. major*. This inhibition was only observed in the presence of PMA indicating a PKC-dependent pathway effect. Altogether, our results suggested that *L. tropica* is more potent in inhibiting PMN O_2_^−^ production than *L. major* but the precise mechanisms associated with NADPH oxidase modulation need to be further explored.

It is well known that phosphorylation of p47^phox^ on several serine residues, especially Ser328, is crucial for NADPH oxidase assembly and activation [36]. Although we observed a phosphorylation on Ser328 of p47^phox^, upon PMN infection with promastigotes, this was not associated with O_2_^−^ production, suggesting that other processes are probably missing for full NADPH oxidase activation following infection with live parasites. Furthermore, previous studies have shown that phosphorylation of p47^phox^ on Ser345 is a key event for the priming process, as it allows prolylisomerase Pin1 binding, which then acts as a molecular switch to facilitate the assembly of NADPH oxidase in human PMN [25,31]. However, in the present study, *L. major* promastigote did not affect Ser345 phosphorylation on p47^phox^. Thus, it may induce NADPH oxidase priming through other mechanisms such as an increase in intracellular calcium or lipids messengers, such as arachidonic acid or phosphatidic acid, thereby favoring the assembly of the enzyme [21]. This effect could also include phosphorylation of p47^phox^ on other sites, translocation of p47^phox^ and/or p67^phox^ to the membrane, fusion of specific granules to the phagosome, or activation of the small G protein Rac 2. Moreover, only live parasites were able to induce p47^phox^ phosphorylation since their respective SLAs did not.

Regarding *L. major,* our observation is consistent with previous studies indicating that *L. major* interaction with PMNs did not elicit the production of reactive oxygen species [37]. This inability to induce NADPH oxidase activation was related to the lack of fusion of tertiary and specific granules of neutrophils with *Leishmania*-containing phagosomes. These granules indeed contain gp91^phox^ and p22^phox^ which are involved in superoxide anion generation. On the contrary, other studies have shown that *L. major* promastigotes are able to induce ROS production in human PMNs [34,35]. Thus, RicciAzevedo et al. observed an increase in ROS production in *L. major* infected human PMNs compared to uninfected cells [33]. In these studies, ROS production was measured by luminolamplified chemiluminescence, a more sensitive approach than the cytochrome C reduction assay used in our study. This may account for the observed difference. Discrepancies may be related to *Leishmania* strains, since we used primary isolates of patients living in Morocco, and therefore differences as interspecific and intraspecific polymorphisms of *Leishmania* proteins were described [38,39].

In addition, SLAs from both strains were neither able to inhibit fMLF-induced p47^phox^ phosphorylation, although they inhibited PMA-induced p47^phox^ phosphorylation. It is known that p47^phox^ could be phosphorylated by several PKC isoforms, and that fMLF and PMA differentially activate these isoforms [30,40]. The difference observed between SLA *versus* live parasite could be consistent with a general observation that antigens released such as exosomes, differ in terms of promastigote properties [41], that have been reported to be increased following a temperature shift mimicking the parasite’s entry into mammalian cells. Similarly, live and dead parasites induced distinct outcomes in infected host cells [42,43]. Therefore, the difference observed between *L. major* and *L. tropica* could be related to a difference in exosome release between these two *Leishmania* strains. However, this cannot exclude the role of additional factors such as lipophosphoglycan (LPG), that strongly inhibits the activity of PKC [44], and is therefore more potent to inhibit PMA than fMLF-mediated p47^phox^ phosphorylation. Because LPG is polymorphic, particularly LPG from *L. tropica*, which is the most complex structure [39], the role of LPG in modulating PMN activation should be further analyzed. ROS production is well-known to contribute in the control of intracellular parasites. Recently, Pitale DM et al. reported that ROS production mediated by *L. major* induces PMN autophagy, which could influence parasite clearance [45]. Our study showed that *L. major* primed ROS production while *L. tropica* impaired it in PMNs, a fact that could lead to a significant difference between the two strains regarding disease outcome. The lesions due to *L. tropica* are usually single, and progress slowly to healing, over several months. In contrast, lesions due to *L. major* are often multiple, and heal after four to six months [15]. A recent analysis of CL cases diagnosed in Casablanca (Morocco) showed heterogeneity in the appearance of lesions that was not significantly associated with either of the responsible *Leishmania* species, i.e., *L. major* and *L. tropica* [46]. This polymorphic clinical appearance of lesions depends on the immune status of the host, the parasite genotype and burden.

Human infection caused by *L. tropica* seems to be more insidious compared to *L. major* infection. Furthermore, there have been reports of the chronic tendency of *L. tropica* CL as well as the differences in disease progression [47]. In particular, by performing transcriptional profiles of lesions caused by either *L. major* or *L. tropica*, we found that the latter presents an aggravated inflammatory/cytotoxic profile [48]. Even if dermal lesions of CL heal spontaneously, the time of healing for *L. tropica* can be as long as 12 months or more. A we know that this species is mainly considered as anthroponotic and that it is responsible in some countries for *Leishmaniasis recidivans* or even sporadically for visceral leishmaniasis, it is important to understand the pathophysiology of CL.

The increase in ROS production dampens infection establishment while the impairment in ROS production would favor infection by allowing greater survival of the parasites within neutrophils, thus facilitating their subsequent transfer into macrophages [9]. Additionally, we have previously reported that *L. tropica* is able to inhibit NO production by macrophages [15], therefore suggesting a more global impact of *L. tropica* on innate immune cells. It seems that *L. tropica* has developed a more efficient escape mechanism of innate immunity than *L. major*. Thus, it is important to understand the pathophysiology of CL going through the study of innate cells and their microbicidal power for a better control of this infection. Presently, the first choice drugs for leishmaniasis chemotherapy are pentavalent antimonials that are significantly toxic and have reported drug resistance, thus reinforcing the need for new therapeutic alternatives.

## 5. Conclusions

To the best of our knowledge, this is the first report addressing the impact of primary moroccan *Leishmania* species on human PMN activity especially on superoxide anions production. ROS production by PMNs is important to control several pathogens, including *Leishmania* parasites. Overall, our results indicate that *L. major* and *L. tropica* modulated O_2_^−^ production by human PMNs differently and independently from p47^phox^ phosphorylation.

Although the precise mechanism of this effect remains to be assessed, our findings may have potential implications concerning the pathogenesis of this parasite. Indeed, PMNs are among the first innate cells that are massively recruited at the bite site and take up the parasite. It is possible that there could be a threshold of infection level required to stimulate ROS production by PMNs, and/or that parasites may prevent ROS generation by some PMNs. Further study on the mechanisms involved in the PMNs responses triggered by these species need to be developed. Likewise, the effect of these species on NADPH activation also needs more investigation. Although this observation has not been extended to additional primary *Leishmania* strains, and the mechanism used by these species to inhibit O_2_^−^ production need to be addressed, we believe that inhibition of PMN killing functions would be an escape mechanism and a strategy to persist into the host providing support for the chronicity and spreading of *L. tropica* in Morocco. Indeed, our previous work demonstrated that this *L. tropica* strain also inhibited NO production by macrophages. We hypothesize that infected PMNs responding to *Leishmania* strain phagocytosis with low or absent ROS production could promote the maintenance of a parasite subset that establishes infection. Furthermore, responses of some infected and bystander PMNs may contribute to a local inflammatory environment that is ineffective at parasite clearance.

Therefore, understanding the mechanisms used by some *Leishmania* spp. to block the neutrophil oxidative burst would be helpful in the development of therapeutic strategies against leishmaniasis.

## Data Availability

Data is contained within the article or Supplementary Materials.

## Supplementary Materials

The following are available online at https://www.mdpi.com/article/10.3390/microorganisms9051025/s1. Figure S1: Factorial analyses for ROS production, Figure S2: Factorial analyses for mechanisms on P47-Phox NADPH oxidase.
