# Supplementary material for: The Modulation of NADPH Oxidase Activity in Human Neutrophils by Moroccan Strains of Leishmania major and Leishmania tropica Is Not Associated with p47phox Phosphorylation
Source: Microorganisms. 2021 May 10;9(5):1025. doi: 10.3390/microorganisms9051025 (PMC8151549; doi:10.3390/microorganisms9051025)

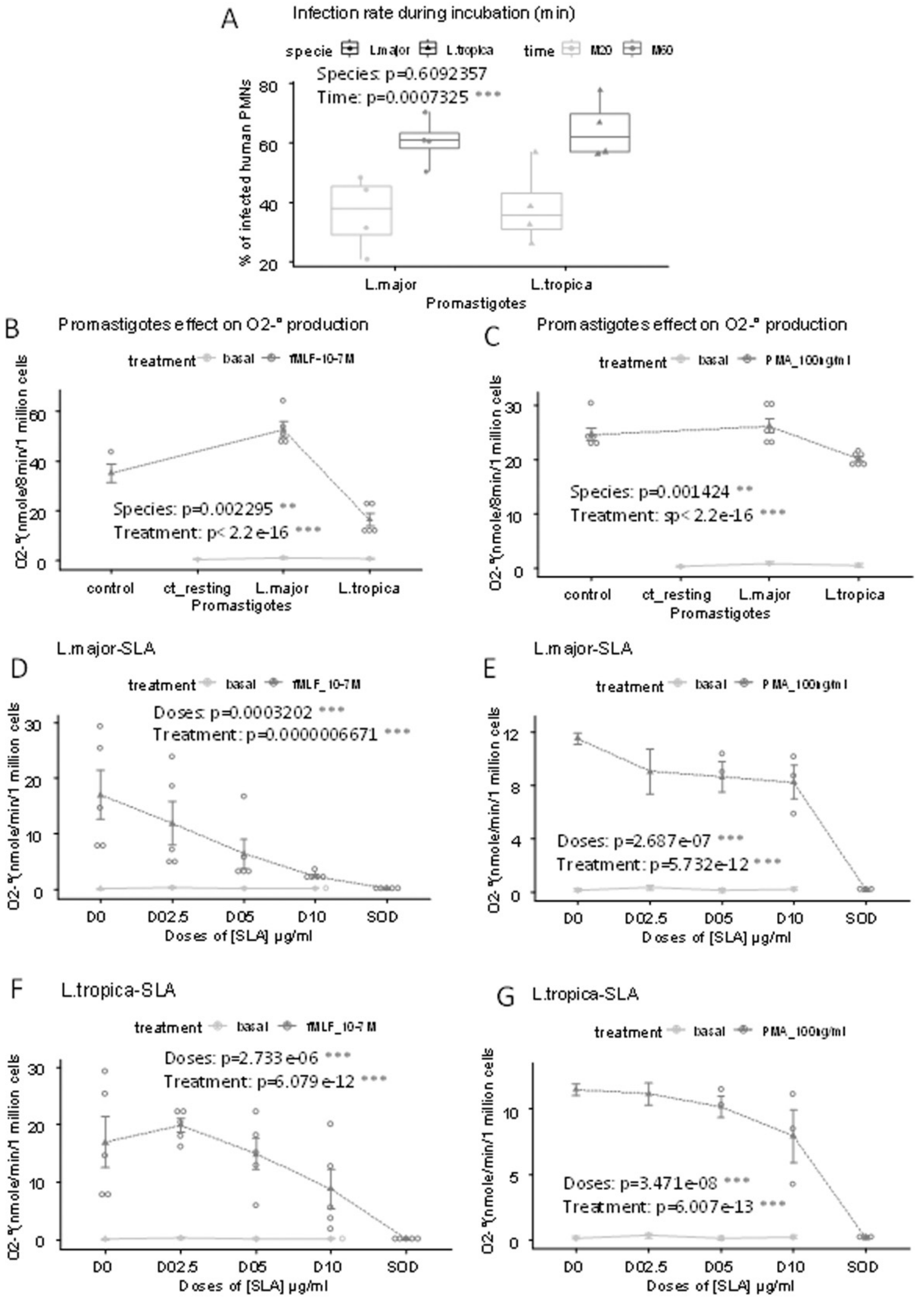

Supplemental Figure 1: Factorial analyses for ROS production: for A to G: p-values on respective factors were obtained by two-way factorial ANOVA; A/Boxplot of PMNs infection rate during incubation in minutes; B/ Dot and Whisker plot of promastigote effect on O2-° radical production in presence of fMLF treatment; C/ Dot and Whisker plot of promastigote effect on O2-° radical production in presenceof PMA treatment; D/ Dot and Whisker plot of L.major-SLA effect on O2-° radical production in presence of fMLF treatment; E/ Dot and Whisker plot of L.major-SLA effect on O2-° radical production in presence of PMA treatment; F/ Dot and Whisker plot of L.tropica-SLA effect on O2-° radical production in presence of fMLF treatment; G/ Dot and Whisker plot of L.tropica-SLA effect on O2-° radical production in presence of PMA treatment

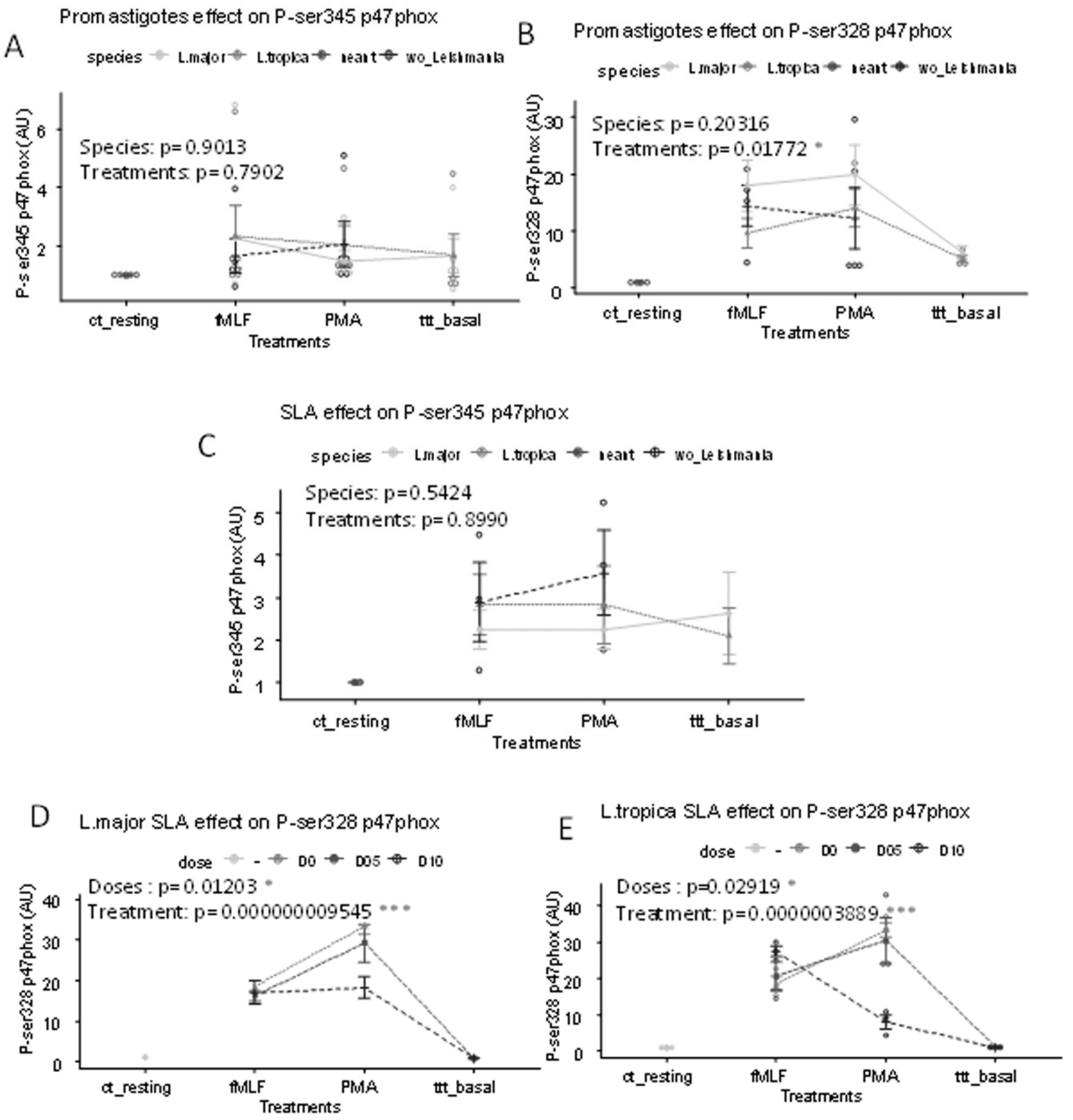

Supplemental Figure 2: Factorial analyses for mechanisms on P47-Phox NADPH oxidase: for A to E: p-values on respective factors were obtained by two-way factorial ANOVA; A/Promastigote effect on Ser345 phosphorylation of p47-phox; A/Promastigote effect on Ser328 phosphorylation of p47-phox; C/ SLA effect on Ser345 phosphorylation of p47-phox; D/L.major SLA effect on Ser328 phosphorylation of p47-phox; E/ L.tropica SLA effect on Ser328 phosphorylation of p47-phox.

Supplementary result (Figure 3'A,B,C,D)

(MAKSOURI et al.)

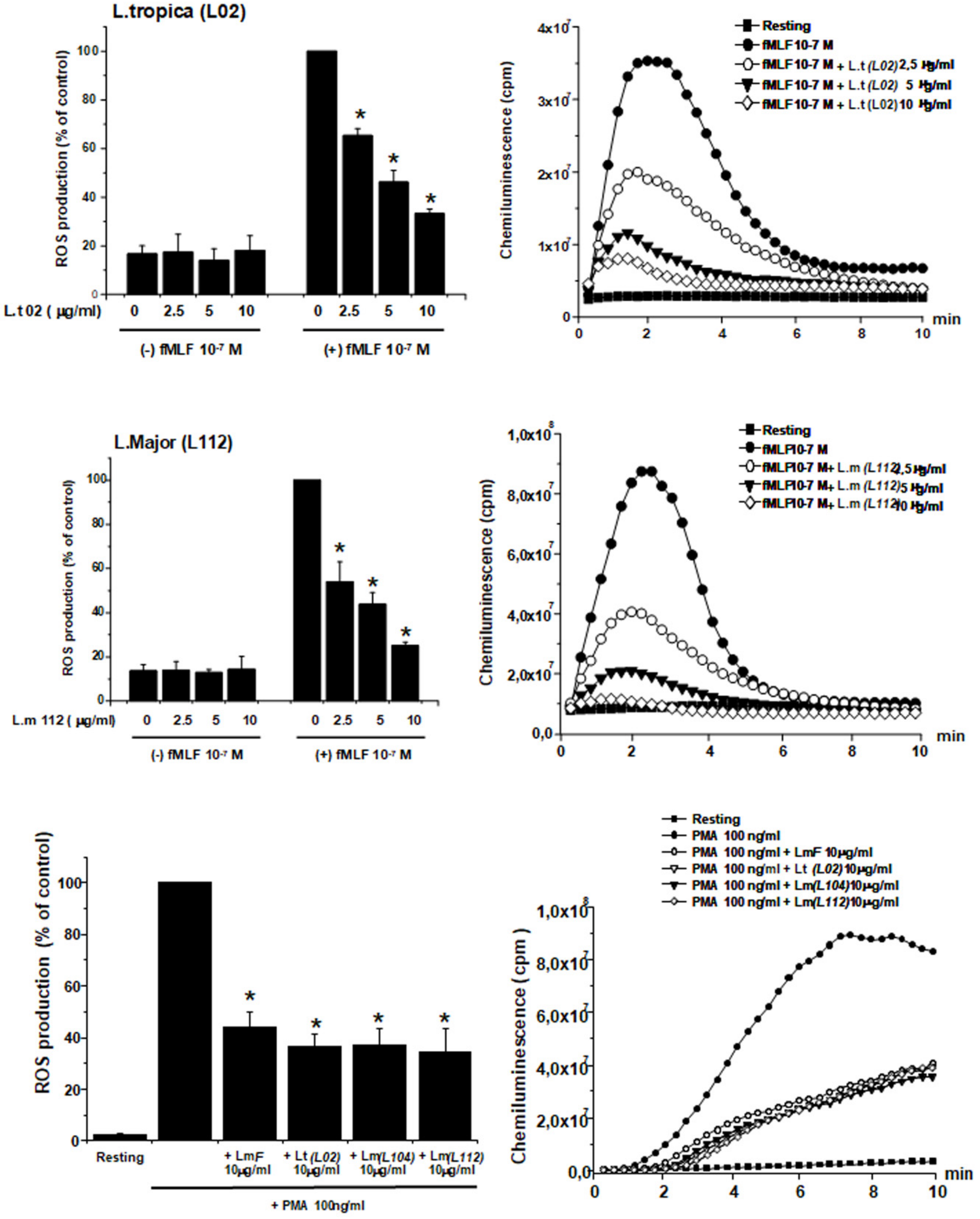

Supplement: Supplementary file 1 [file microorganisms-09-01025-s001.zip › microorganisms-1147192-supplementary.pdf]
